# Efficacy of Oxy-Ionic Solutions With Varying pH Levels Against Streptococcus mutans In Vitro

**DOI:** 10.7759/cureus.61025

**Published:** 2024-05-24

**Authors:** Xu Ming, Rozita Hassan, Suharni Mohamad, Wan Nazatul Shima Shahidan

**Affiliations:** 1 Orthodontic Unit, School of Dental Sciences, Universiti Sains Malaysia, Kelantan, MYS; 2 Molecular Biology Unit, School of Dental Sciences, Universiti Sains Malaysia, Kelantan, MYS; 3 Pharmacology Unit, School of Dental Sciences, Universiti Sains Malaysia, Kelantan, MYS

**Keywords:** antimicrobial, oxidizing solution, mouthwash, dental caries, streptococcus mutans

## Abstract

Background

Chlorhexidine (CHX) is a widely used antimicrobial agent known for its ability to inhibit cariogenic bacteria, reduce plaque formation, neutralize acidity, and promote remineralization. However, the effectiveness of oxy-ionic solutions at different pH levels as an alternative antimicrobial treatment requires further exploration. This study aims to compare the antimicrobial effects of oxy-ionic solutions at various pH levels with those of CHX and fluoride.

Methodology

This study employed disc diffusion tests to measure the inhibition zone diameters of each solution and broth dilution assays to determine the minimum inhibitory concentration (MIC) and minimum bactericidal concentration (MBC).

Results

The oxy-ionic solutions exhibited varying degrees of antimicrobial effectiveness depending on their pH levels. The solution at pH 5 demonstrated the best antimicrobial performance among the oxy-ionic solutions, with inhibition zones comparable to those of CHX. The MIC and MBC values indicated that oxy-ionic solutions in mildly acidic environments generally resulted in better antimicrobial activity.

Conclusions

The study concludes that while CHX remains highly effective against cariogenic bacteria, oxy-ionic solutions, particularly at pH 5, offer a promising alternative. The antimicrobial efficacy of oxy-ionic solutions is influenced by their pH levels. Further research is recommended to explore the long-term effects and clinical applications of oxy-ionic solutions in maintaining oral health and preventing disease.

## Introduction

Dental caries is a common chronic infectious disease in the oral cavity caused by bacteria, characterized by hard tissue destruction of teeth. It occurs at any age but is more prevalent in children and is closely related to oral microbiota [1]. Consuming excessive carbohydrates can lead to tooth decay by promoting acid production and disrupting the saliva’s buffering capacity. The microbial shift in oral biofilms from good to bad promotes enamel loss and demineralization of mineral hydroxyapatite crystals [2].

The formation of cariogenic biofilms is a dynamic process dominated by acid-producing and acid-tolerant bacteria, notably *Streptococcus mutans* [3]. Their ability to produce high levels of acid, withstand acidic environments, and form robust biofilms in the presence of sucrose marks them as key culprits in dental caries development [4]. Moreover, the co-infection of *Streptococcus mutans* is linked to persistent oral infections. Such infections tend to exacerbate caries prevalence in children and increase the likelihood of recurrence post-clinical intervention [5,6].

Oxy-ionic solution is a strong oxidizing solution that can be produced in the pH range of 2.5 and 8.5, but for most applications, it is produced in the neutral pH range of 6.5 to 7.2. Oxy-ionic is the most common type of ion in the world. With an oxidation-reduction potential (ORP) of between +600 and +1,200 mV, it is an effective fungicide that can be used as a broad-spectrum fungicide to kill all types of microorganisms, including viruses, fungi, and bacteria. Escherichia coli logarithm can be reduced by 8 in 10 seconds. For more than a decade, the daily use of oxy-ionic solution as a disinfectant for hard surfaces has proven that microorganisms do not gain immunity to them. The antimicrobial efficacy of oxy-ionic solutions is largely affected by pH, which, in turn, affects its ORP. It is known that lower pH values correspond to higher ORP values, increasing the microbial killing efficiency of these solutions. This feature is especially important in dental care applications, where effective pathogen control is critical to preventing infection and maintaining oral health [7].

Existing treatments for dental caries include removing and filling the decayed tissue, using fluoride toothpaste and mouthwash, and, in recent years, researchers have been studying alternative treatment strategies aimed at addressing dental caries and periodontal disease. Among these methods, there is great interest in the use of chlorhexidine (CHX), which prevents dental caries by inhibiting the growth of cariogenic bacteria, reducing plaque formation, neutralizing acid, and promoting remineralization. Despite its efficacy, CHX has its limitations and potential side effects that must be considered. However, its disadvantages, including altered taste, tooth discoloration, and xerostomia, must be carefully considered [8].

The oxy-ionic solution has shown potential for promoting antimicrobial activity and tissue regeneration. However, there is a lack of comprehensive understanding regarding the antimicrobial efficacy of oxy-ionic solutions against *Streptococcus mutans* [9,10].

In this study, we aim to bridge this knowledge gap by investigating the antimicrobial efficacy of oxy-ionic solutions at various pH levels against *Streptococcus mutans*.

## Materials and methods

One cryovial *Streptococcus mutans* (ATCC® 25175™) was procured from the Craniofacial Science Laboratory at the School of Dental Sciences, Universiti Sains Malaysia. Oxy-ionic solutions of varying pH levels (one bottle of each pH value), certified with ISO 9001 Quality Certification, were supplied by Yusenviro Sdn Bhd.

Ph measurement

The pH of the oxy-ionic solution was measured and recorded using the HANNA pH 211 microprocessor pH meter. Three samples from each solution were tested. The pH meter was calibrated before use.

Microbial culture

The experimental methodology commenced with the revival of *Streptococcus mutans* from glycerol stocks [11]. * Streptococcus mutans* was streaked onto Mueller-Hinton agar [12]. The plates were then incubated for 48 hours at 37°C in a 5% CO_2_ atmosphere. After incubation, three to five colonies were harvested and resuspended in a growth medium. These suspensions were vortexed to ensure homogeneity before adjusting turbidity to meet the 0.5 McFarland standard (equivalent to approximately 1.5 × 10^8^ CFU/mL). This adjustment is accurately measured using a densitometer to ensure the accuracy of subsequent analysis [13]. *Streptococcus mutans* were grown anew for each independent experiment.

Antimicrobial testing

Disk Diffusion Testing

Initially, sterilized swabs were saturated with *Streptococcus mutans* and excess liquid was carefully removed by pressing the swab against the inner edge of the test tube. The swab was then used to streak Mueller-Hinton agar to ensure even distribution of the bacteria. The Mueller-Hinton agar plates with *Streptococcus mutans* were allowed to rest for two to five minutes to absorb moisture. Subsequently, paper discs soaked in alcohol-free CHX, fluoride mouthwash, oxy-ionic solutions of varying pH, and distilled water were applied to the agar. Among them, CHX and fluoride mouthwash served as the positive control group, and distilled water served as the negative control group. The diameter of the inhibition zones around each disc was meticulously measured from three different directions using calipers, and these measurements were averaged to determine the final efficacy. All tests were performed in triplicate [14].

Broth Dilution Method

The sensitivity of *Streptococcus mutans* to varying pH levels of oxy-ionic solutions was assessed using the agar dilution method, as per Clinical & Laboratory Standards Institute guidelines [15]. In a biosafety cabinet, a series of dilutions were prepared in 12 sterile centrifuge tubes, starting with 1,000 μL of oxy-ionic solution in the first tube and serial dilutions down to 0.05% concentration in the 12th tube. For the assay, 100 μL from each tube was transferred to wells A1 through A12 of a 96-well plate, which contained a bacterial suspension of 1 × 10^6^ cfu/mL of *Streptococcus mutans*. After incubating the plate at 37°C for 24 hours, resazurin was added to assess bacterial growth by observing color changes in the wells. Wells without color change indicated concentrations above the minimum inhibitory concentration (MIC). For minimum bactericidal concentration (MBC) determination, 10 μL from wells showing no growth was subcultured and incubated for an additional 24 hours. The absence of growth indicated the MBC of the oxy-ionic solution. CHX, fluoride, and distilled water were used as controls to ensure that there were no operational errors in the experiment. This procedure was replicated three times [16].

## Results

pH analysis

SPSS software (IBM Corp., Armonk, NY, USA) was used to conduct descriptive statistical analysis of the experimental data. The results showed that the data were within the 95% confidence interval. All types of oxy-ionic solutions met their labeled specifications, with a pH ranging from 3.44 to 7.38, covering acidic to neutral conditions. The fluctuations within this range remained within acceptable limits. For more details, refer to Table 1.

**Table 1:** Measured pH values of oxy-ionic solutions.

Solution description	Expected pH	Measured pH	95% confidence interval	
			Lower limit	Upper limit
Oxy-ionic solution (pH 3)	3	3.24 ± 0.40	3.14	3.34
Oxy-ionic solution (pH 5)	5	5.18 ± 0.72	5.00	5.36
Oxy-ionic solution (pH 7)	7	7.05 ± 0.75	6.86	7.24

Antimicrobial analysis

Disk Diffusion Testing Analysis

The oxy-ionic solution showed antimicrobial activity against *Streptococcus mutans*, as shown in Table 2.

**Table 2:** Inhibition zone diameter of Streptococcus mutans in different solutions. CHX = chlorhexidine

Solutions	Inhibition zone diameter (mm)		95% confidence interval		Shapiro-Wilk		
	Microbes	Mean ± SD	Lower limit	Upper limit	Statistic	df	Sig.
Oxy-ionic (pH 3)	*Streptococcus mutans*	10.14 ± 0.24	9.960371795	10.32407265	0.953	9	0.719
Oxy-ionic (pH 5)	*Streptococcus mutans*	12.63 ± 0.18	12.49294401	12.76261154	0.909	9	0.309
Oxy-ionic (pH 7)	*Streptococcus mutans*	7.45 ± 0.10	7.369799862	7.523533471	0.915	9	0.352
CHX	*Streptococcus mutans*	27.30 ± 0.08	27.26028857	27.38193365	0.953	9	0.721
Fluoride	*Streptococcus mutans*	7.30 ± 0.04	7.2775	7.3314	0.945	9	0.633

To evaluate the antibacterial effects of oxy-ionic solutions, CHX, and fluoride with different pH values ​​on *Streptococcus mutans*, we conducted a disk diffusion experiment. SPSS software was used to conduct descriptive statistical analysis and the Shapiro-Wilk normality test on the experimental data. The results showed that the data were normally distributed and were within the 95% confidence interval. Experimental results showed that the antibacterial effect of oxy-ionic solutions on *Streptococcus mutans* increased as the pH value decreased. Specifically, at pH 3 and 5, the oxy-ionic solution showed larger average inhibition zone diameters (IhDs) against *Streptococcus mutans* compared to fluoride mouthwash, indicating superior antimicrobial efficacy.

The oxy-ionic solution showed optimal antimicrobial efficacy against *Streptococcus mutans* at pH 5, with a mean IhD of 12.63 mm and a standard deviation of 0.18. As the pH value increased toward neutrality, the inhibitory effect on mutant yeast diminished; though not as markedly as in acidic conditions, it still maintained some inhibitory activity.

As can be seen in the data in Table 3, in experiments with CHX as the control group, the IhD for *Streptococcus mutans* in the control group was measured at 27.3 ± 0.08 mm. In the experimental groups treated with oxy-ionic solutions at pH 3, pH 5, and pH 7, the IhD values were 10.1 ± 0.24 mm, 12.63 ± 0.18 mm, and 7.45 ± 0.10 mm, respectively. Due to the failure to meet the assumption of homogeneity of variances, Welch’s test was performed, resulting in an F-value of 75,063.34 and a p-value of 0.05, indicating statistically significant differences in IhD values among the four groups. Subsequent pairwise comparisons using the Tamhane method revealed that the IhD for *Streptococcus mutans* was highest in the CHX control group, followed by the groups treated with oxy-ionic solutions at pH 5, pH 3, and pH 7, respectively, with significant differences observed between the groups.

**Table 3:** ANOVA table with CHX as the control group. Different letters indicate statistically significant differences between groups (p < 0.05). ANOVA = analysis of variance; CHX = chlorhexidine

Microbes	Inhibition zone diameter (mm)				F value	P-value
	CHX	Oxy-ionic (pH 3)	Oxy-ionic (pH 5)	Oxy-ionic (pH 7)		
*Streptococcus mutans*	27.3 ± 0.08 ^a^	10.14 ± 0.24 ^c^	12.63 ± 0.18 ^b^	7.45 ± 0.10 ^d^	75,065.34	0.000

As shown in Table 4, in the experiments where fluoride served as the control group, the IhD for *Streptococcus mutans* was recorded at 7.3 ± 0.04 mm in the control setting. For the experimental groups that received oxy-ionic treatments at pH 3, pH 5, and pH 7, the IhD measurements were 10.14 ± 0.24 mm, 12.63 ± 0.18 mm, and 7.45 ± 0.10 mm, respectively. The assumption of homogeneity of variances was not met, prompting the application of Welch’s test which resulted in an F-value of 2,772.772 and a p-value of <0.001, indicating significant statistical differences in IhD values across the groups. Follow-up pairwise comparisons using the Tamhane method indicated the highest IhD for *Streptococcus mutans* in the group treated with oxy-ionic solutions at pH 5 and pH 3. The lowest IhD values were found in the oxy-ionic solution at pH 7 and the fluoride control groups, with no statistically significant difference noted between them.

**Table 4:** ANOVA table with fluoride as the control group. Different letters indicate statistically significant differences between groups (p < 0.05). ANOVA = analysis of variance

Microbes	Inhibition zone diameter (mm)				F value	P-value
	Fluoride	Oxy-ionic (pH 3)	Oxy-ionic (pH 5)	Oxy-ionic (pH 7)		
*Streptococcus mutans*	7.3 ± 0.04 ^c^	10.14 ± 0.24 ^b^	12.63 ± 0.18 ^a^	7.45 ± 0.10 ^c^	2,772.772	0.000

Overall, the oxy-ionic solution demonstrated effective antimicrobial activity against *Streptococcus mutans* under acidic conditions, particularly showing the strongest activity at pH 5. However, as pH approached neutrality, its antimicrobial effect weakened. This change may be attributed to variations in the chemical composition of the oxy-ionic solution at different pH values, leading to an impact on microbial cell walls.

Broth Dilution Method Analysis

To test the MIC and MBC of oxy-ionic at different pH values, a broth dilution test was performed. Descriptive statistical analysis and the Shapiro-Wilk test of the experimental data were performed using SPSS software. The results showed that the data obeyed a normal distribution and were within the 95% confidence interval. As shown in Table 5, under conditions of pH 3, the oxy-ionic solution exhibited significant antimicrobial activity against *Streptococcus mutans*, with the MIC at 25% and the MBC at 50%. This indicates that, even in highly acidic environments, the oxy-ionic solution effectively inhibited bacterial growth and possessed bactericidal properties. As the pH value increased to 5, the antimicrobial efficacy of the oxy-ionic solution against the bacteria decreased, with both MIC and MBC rising to 50%. This may suggest that as the pH approached neutrality, changes in the charge state or stability of the active components within the oxy-ionic solution occurred, thereby affecting their interaction with the bacterial cell wall and penetration capabilities. At a pH of 7, where conditions were nearly neutral, the MIC and MBC for the bacteria reached their highest at 100%. This indicated a significant reduction in the antimicrobial effectiveness of the solution against *Streptococcus mutans*.

**Table 5:** The MIC and MBC of oxy-ionic solutions with varying pH levels against Streptococcus mutans. + = bacterial growth; - = no bacterial growth; MIC = minimum inhibitory concentration; MBC = minimum bactericidal concentration

Solutions	*Streptococcus mutans*											
	100%	50%	25%	12.5%	6.25%	3.12%	1.56%	0.78%	0.39%	0.20%	0.10%	0.05%
Oxy-ionic (pH 3)	-	-	-	+	+	+	+	+	+	+	+	+
Oxy-ionic (pH 5)	-	-	+	+	+	+	+	+	+	+	+	+
Oxy-ionic (pH 7)	-	+	+	+	+	+	+	+	+	+	+	+

The research findings demonstrate a close correlation between the antimicrobial efficacy of the oxy-ionic solution and environmental pH values. Under acidic conditions, particularly at pH 3 and 5, the solution exhibited strong inhibitory and bactericidal effects against the tested microorganisms. However, as the pH increased, its antimicrobial effectiveness gradually diminished. This phenomenon may be related to the ionization level, stability, and interaction of active components in the solution with bacterial cell walls at different pH values.

## Discussion

The antimicrobial efficacy of oxy-ionic solutions is significantly influenced by the pH of the environment [17]. The results help to elucidate the relationship between the pH level and the inhibitory action of oxy-ionic solutions against *Streptococcus mutans* [18]. According to pH analysis, all oxy-ionic solutions conformed to their labeled specifications, ranging from a pH of 3.44 to 7.38, encompassing acidic to neutral conditions. This pH range is maintained within acceptable limits, ensuring the reliability of the solutions for the intended experimental conditions [19].

Under acidic conditions (pH 3), the oxy-ionic solution showed robust antimicrobial activity with an MIC of 25% and an MBC of 50%, indicating that the active components within the solution can exert both inhibitory and bactericidal effects even in a strongly acidic environment. However, as the pH increased toward more neutral values (pH 5 and 7), a decline in antimicrobial potency was observed, with the MIC and MBC values increasing, peaking at 100% at pH 7 [20].

The disk diffusion testing further supported these findings, showing that the oxy-ionic solution exhibited a greater zone of inhibition against *Streptococcus mutans* at pH 5 compared to other tested pH values. This suggests optimal antimicrobial activity in a slightly acidic environment, which aligns with the natural pH of the oral cavity in many pathological conditions. The significant decrease in efficacy at pH 7 highlights the need to optimize the solution’s composition for a broader pH range, considering the pH variations in different oral health conditions [21].

CHX, used as a standard comparative antimicrobial agent, showed the highest inhibitory effect with an average IhD of 27.30 mm, establishing it as a benchmark for antimicrobial effectiveness against *Streptococcus mutans*. Meanwhile, fluoride, often used in dental care products, demonstrated lesser antimicrobial action compared to the oxy-ionic solution (pH 5), underscoring the potential of oxy-ionic solutions as effective antimicrobial agents in dental care, particularly at specific pH conditions.

It is postulated that the changes in antimicrobial efficacy with pH variations are due to the altered ionization states or stability of the solution’s active components, which affect their interaction with bacterial cell walls. As pH approaches neutrality, the ionization level of these components may decrease, reducing their affinity for the negatively charged bacterial cell wall, thus diminishing their penetration and subsequent bactericidal action [21].

Limitations

While the findings of this study provide valuable insights into the antimicrobial efficacy of oxy-ionic solutions across different pH levels, several limitations should be considered. First, the in vitro nature of the experiments may not fully replicate the complex interactions and dynamics present in the human oral cavity. The results obtained from such controlled laboratory settings may not translate directly to clinical outcomes. Second, the study focused solely on the effect of pH on the efficacy of oxy-ionic solutions against *Streptococcus mutans* and did not explore other oral pathogens that contribute to dental diseases. This narrows the scope of the findings and limits their applicability to a broader range of microbial challenges encountered in dental care. Additionally, while the study demonstrates the decline in antimicrobial potency with increasing pH, the specific mechanisms by which pH influences the activity of the active components of oxy-ionic solutions were not directly assessed. More importantly, this study did not take into account the polymicrobial synergy and dysbiosis characteristic of periodontal disease. Periodontal disease is a complex disease caused by a variety of bacteria. Simply studying the antibacterial effect of a single bacterial species may ignore the key synergistic effects. Understanding these mechanisms could provide deeper insights into optimizing the formulation for enhanced effectiveness across a wider pH spectrum.

## Conclusions

The findings substantiate the pH-dependent antimicrobial activity of oxy-ionic solutions against *Streptococcus mutans*, with the most significant effects observed in weak acidic conditions.
